# Separate Spontaneous Extracranial and Intracranial Left Internal Carotid Artery Dissections Causing an Acute Ischemic Stroke: A Case Report

**DOI:** 10.7759/cureus.69777

**Published:** 2024-09-20

**Authors:** Andrew Evans, Jahanzeb Rehan

**Affiliations:** 1 Stroke Medicine, King's Mill Hospital, Sutton-in-Ashfield, GBR

**Keywords:** acute ischaemic stroke, internal carotid artery, non-traumatic carotid artery dissection, stroke in young population, young onset stroke

## Abstract

We present a case of a 45-year-old man who suffered simultaneous intracranial and extracranial dissections within the same artery, ultimately leading to a large left-sided ischemic stroke. To our knowledge, this is the only reported case of multiple dissections of the same head or neck (cervicocephalic) artery. We discuss the current evidence and literature around the characteristics of patients who experience multiple cervicocephalic artery dissections.

## Introduction

In cervicocephalic artery dissections (CAD), vessel wall damage leads to rupture and subsequent hematoma formation within the arterial wall between the tunica intima and tunica media, creating a false lumen. This hematoma may extend over time and cause luminal stenosis or occlusion. Local thrombus formation also occurs, creating the potential for embolism and hence stroke. CAD occurs with an estimated prevalence of up to five cases per 100,000 per year [1]. Considering the entire stroke cohort, it is an uncommon etiology, accounting for around 2% of acute ischemic strokes [2]. Among young and middle-aged adults, however, it has been identified as an important etiology, accounting for up to 25% of cases [3]. CAD can be either intracranial or extracranial and can affect any of the cervicocephalic arteries. Although intracranial dissections are less common, they are much more likely to lead to stroke [4]. In European and American studies, dissections are reported to occur three to four times more commonly in the internal carotid arteries (ICA) than the vertebral arteries (VA) [5]. VA dissections are often intracranial [5], but ICA dissections are much more commonly extracranial and typically do not extend beyond the entry of the artery into the petrous portion of the temporal bone, which is the point of entry into the cranium [6]. CAD affecting more than one artery is reported in around 15-20% of cases, bilateral ICA dissections being the most common [7]. To our knowledge, there have been no reported cases of separate intracranial and extracranial dissections of the same cervicocephalic artery prior to this report. CAD sometimes follows neck trauma, but major trauma is not a necessary precondition; other risk factors that have been identified include migraine, hypertension, and a family history of vascular disease [8]. Evidence based on studies of connective tissue ultrastructure and genetic variations suggests that an underlying, subclinical connective tissue disorder may lead to arterial wall weakness and hence a predisposition to CAD [9].

## Case presentation

A 45-year-old Caucasian man with no significant medical history was brought to our unit following the onset of a severe left-sided headache in the early hours causing him to wake, later followed by the onset of right-sided facial and limb weakness at 11 am. The patient had drunk a large quantity of alcohol the prior evening. He was a heavy smoker of around 20 cigarettes per day and had a history of heavy alcohol consumption. The patient had used cocaine most weekends for the past couple of years, including the night prior to his admission to the hospital. There was no known history or physical signs of head or neck trauma.

At presentation, the patient was found to have dense right-sided weakness and marked right-sided sensory loss and neglect, with moderate dysarthria. The patient’s Glasgow coma score was 15/15, and his National Institutes of Health Stroke Scale at admission was calculated to be 14. The patient was also noted to have a left-sided ptosis and miosis. There were no obvious signs of head or neck trauma.

**Figure 1:**
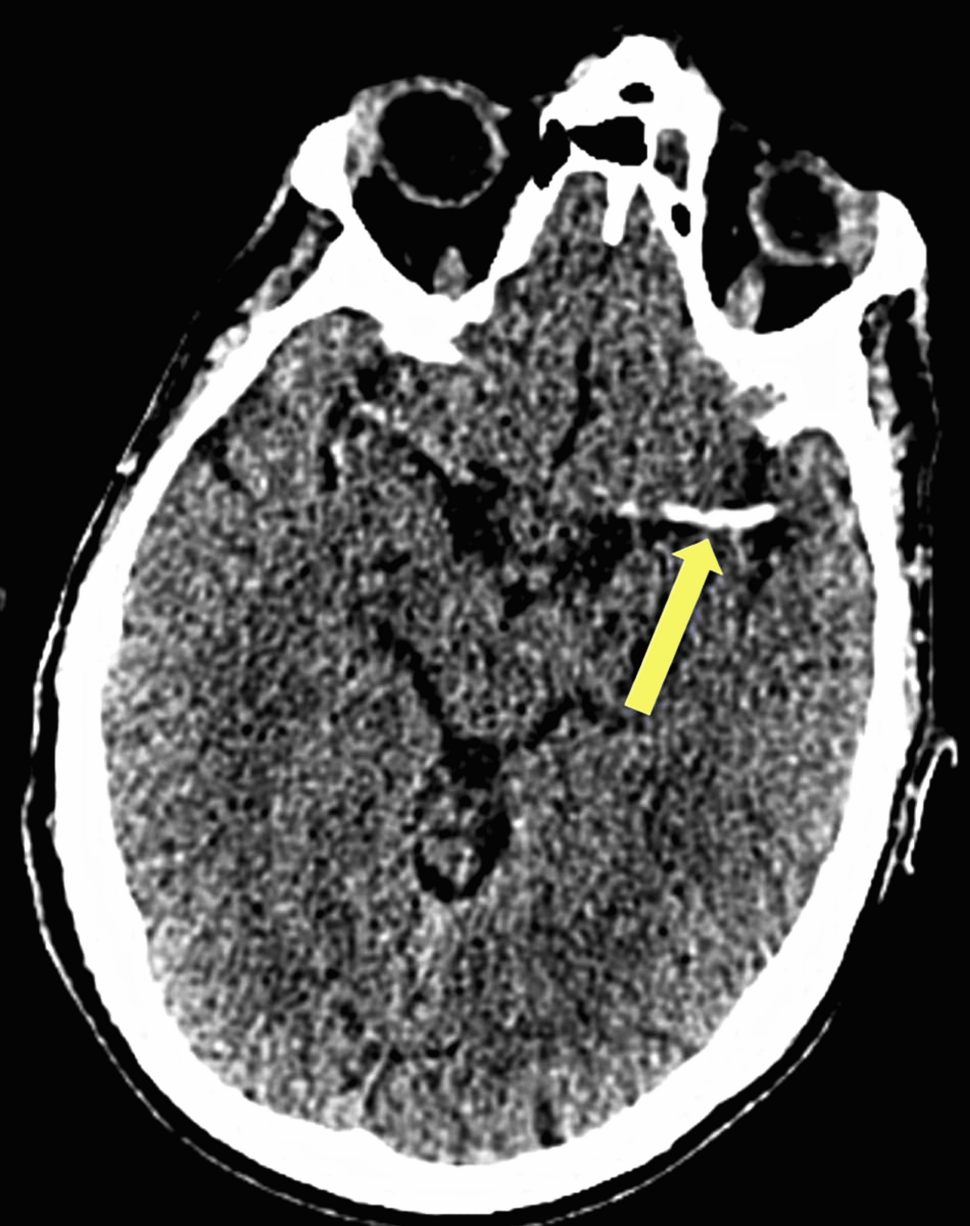
CT brain showing hyperdense left MCA CT: computed tomography, MCA: middle cerebral artery

A CT brain at admission revealed a large left middle cerebral artery (MCA) territory ischemic stroke secondary to occlusion or hyperdensity seen within the left distal supraclinoid ICA, extending along the whole length of the M1 segment of the left MCA to the bifurcation, consistent with an acute thrombus, with possible further thrombus in the left ICA, as seen in Figure 1. The patient consented to intravenous thrombolysis. Following this, he was started on secondary preventative medications. The patient presented at a weekend where there was no provision for immediate mechanical thrombectomy.

**Figure 2:**
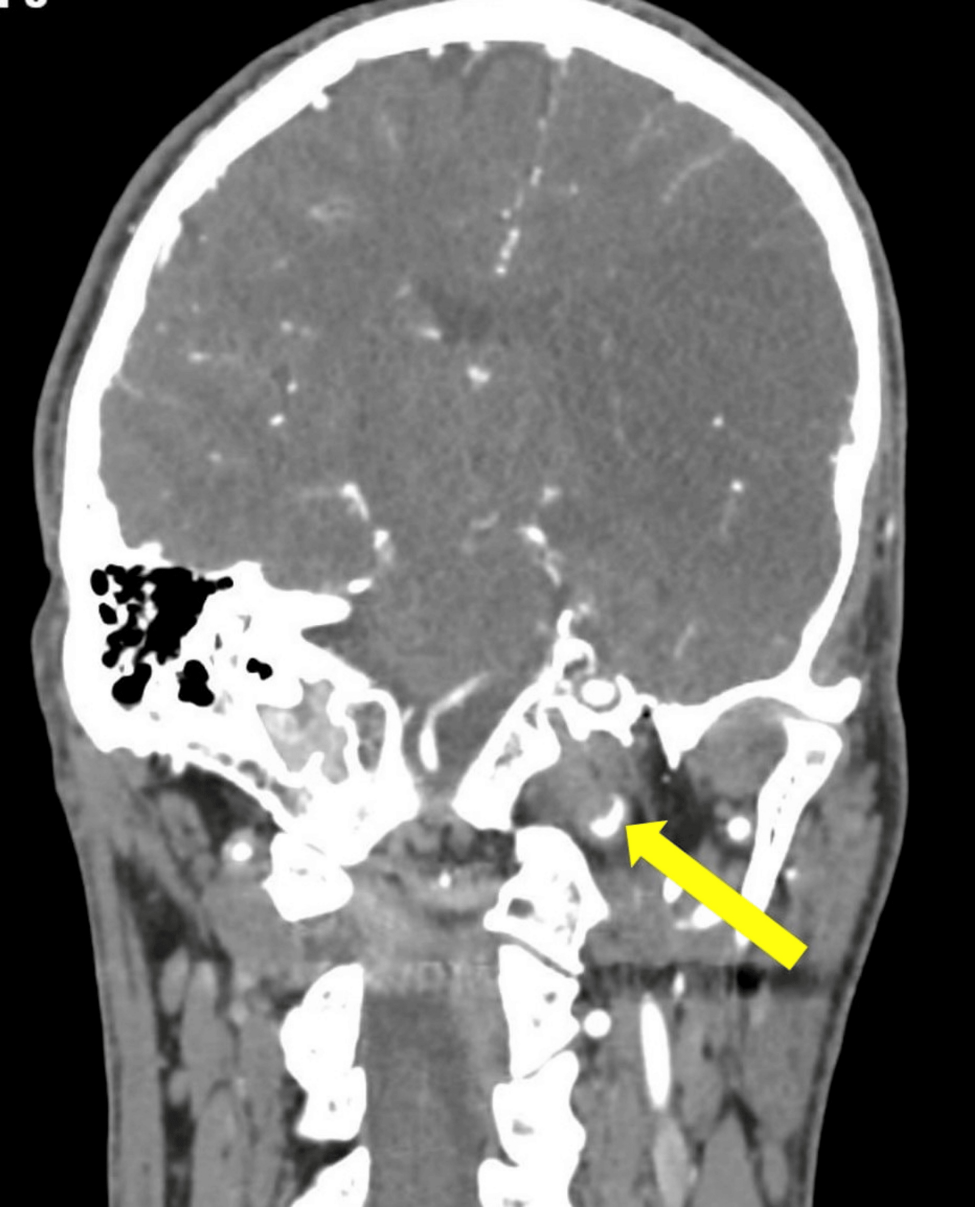
Coronal CT angiogram showing dissection of the cervical part of the left ICA CT: computed tomography, ICA: internal carotid artery

**Figure 3:**
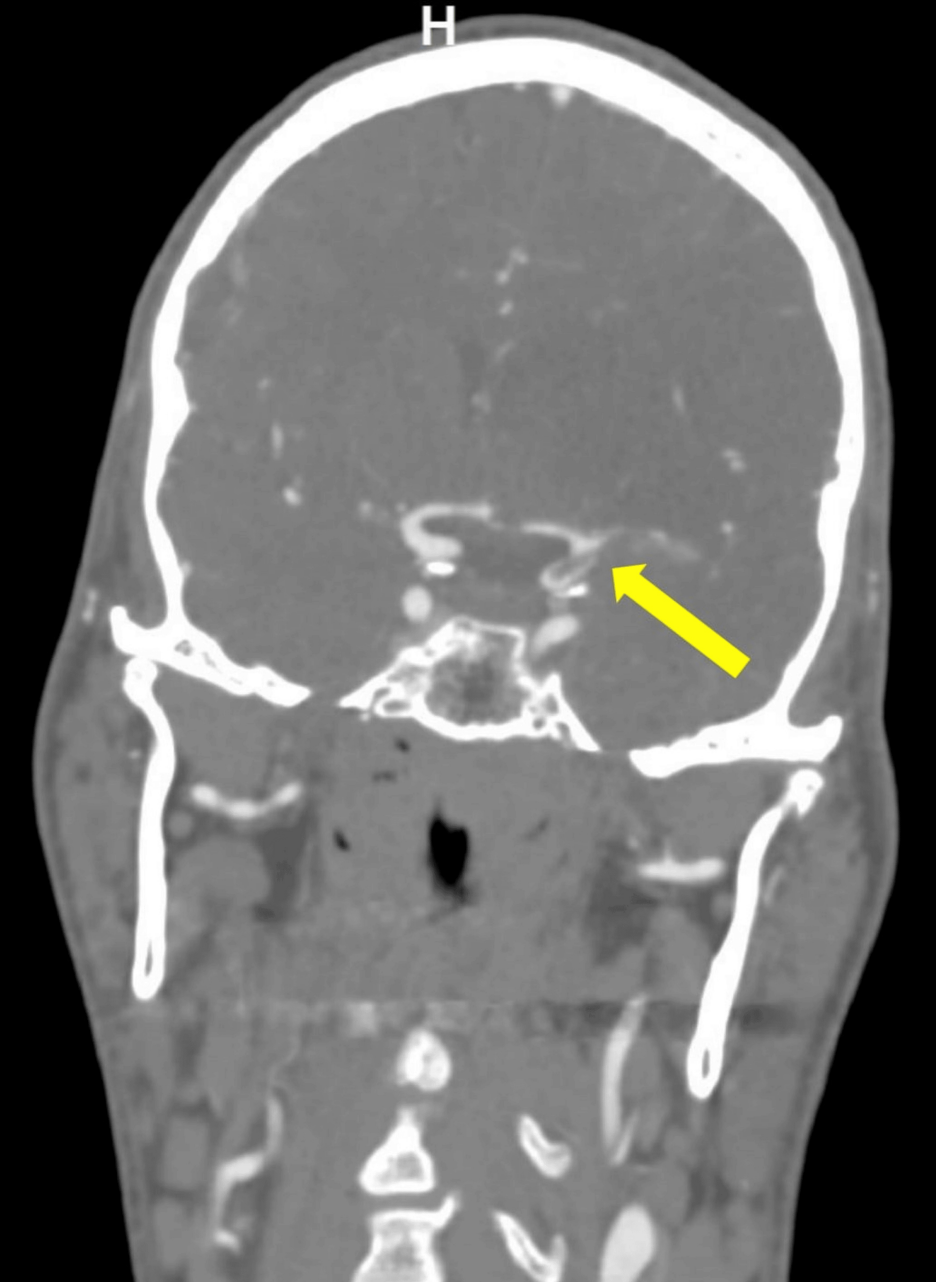
Dissection in the clinoid and supraclinoid parts of the left ICA ICA: internal carotid artery

**Figure 4:**
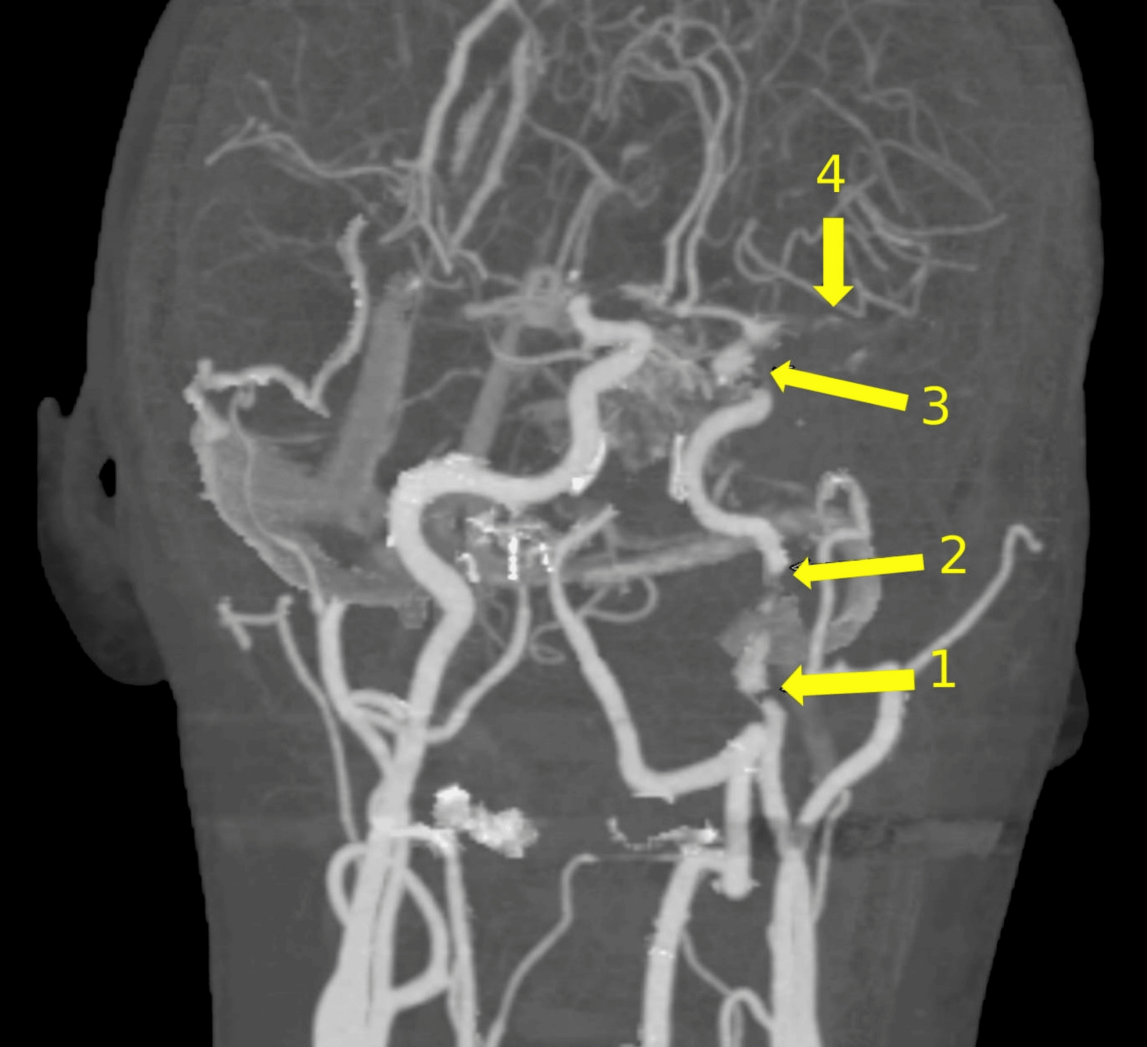
Reconstructed CT angiogram image showing (1) dissection in the distal cervical ICA, (2) refilling of the carotid canal and cavernous part of the left ICA, (3) dissection in the clinoid and supraclinoid ICA, and (4) extension of the dissection in the left MCA

The patient continued to complain of an ongoing sharp, left-sided headache, dissecting the mid-scalp, face, and neck. There were also indications of Horner’s syndrome ipsilateral to the headache. These symptoms alerted the stroke team to the possibility of carotid artery dissection. A CT angiogram revealed a left ICA occlusion just a few centimeters below the skull base in the extra-cranial cervical segment of the ICA, showing true and false lumens, as shown in Figure 2, with refiling within the carotid canal up to another similar occlusion in the clinoid and supraclinoid parts of the ICA, extending to the whole length of the MCA, again with both true and false lumens, as seen in Figure 3. A reconstructed image showing the two separate dissections is shown in Figure 4.

These CT angiogram findings were highly suggestive of separate intracranial and extracranial dissections of the same ICA. The petrous part of the ICA is described as obscure and not imaged well often in the literature, but the refilling within the carotid canal up to a further occlusion is in keeping with two separate dissections.

**Figure 5:**
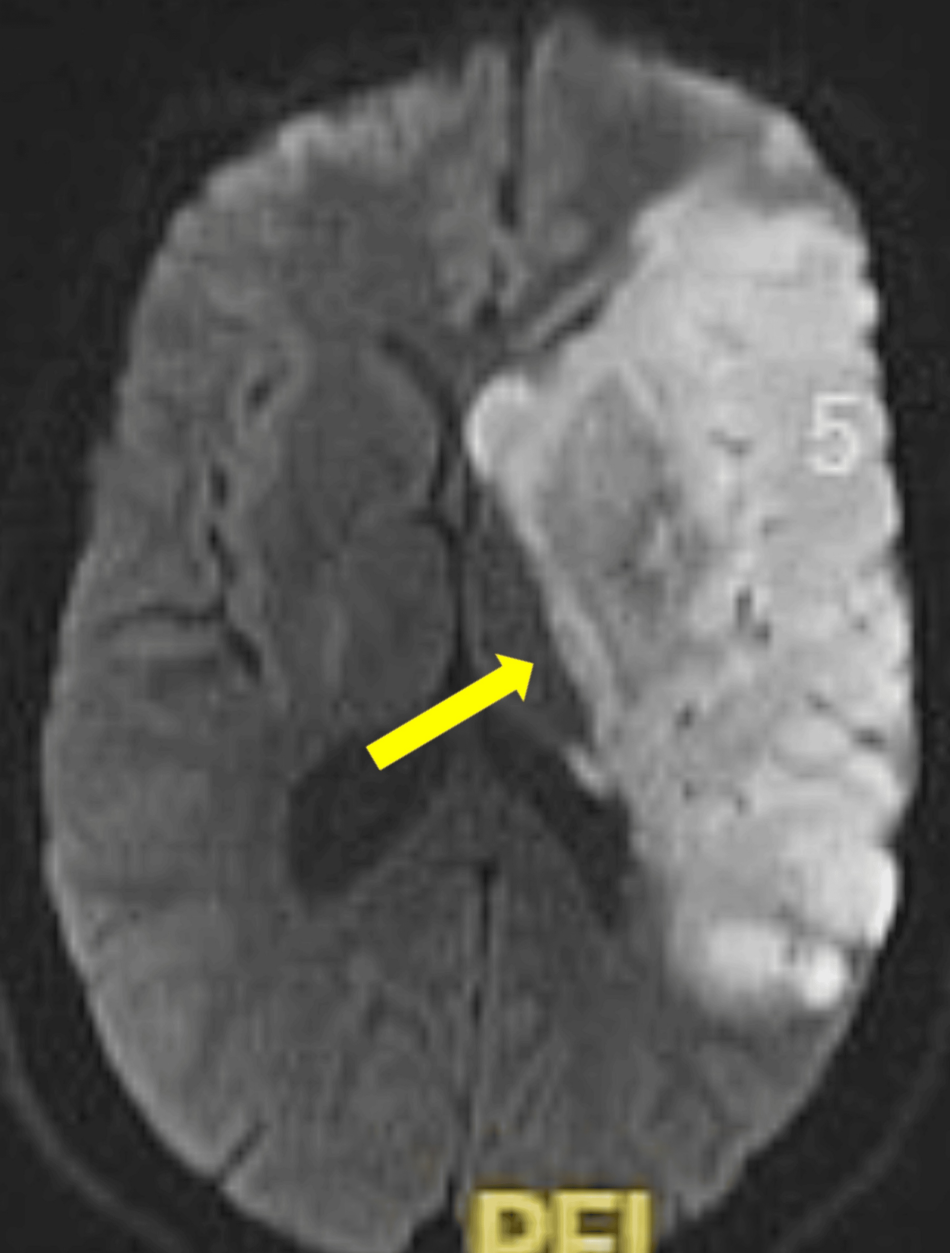
MRI brain showing the left MCA territory ischemic stroke MRI: magnetic resonance imaging, MCA: middle cerebral artery

Mechanical thrombectomy was contraindicated at this time due to the now radiologically well-established stroke, as the service was only available the following day. A later MRI brain confirmed a left MCA territory stroke with diffusion restriction in the left basal ganglia, as seen in Figure 5.

Given the patient’s young age, thrombophilia screening was commenced. The tests performed were ANA, beta-2 glycoprotein, immunoglobulins, anti-cardiolipin antibodies, dsDNA antibodies, lupus anticoagulant, and extractable nuclear antigen screening. The results of this battery of tests were all unremarkable.

This man had a strong history of hemorrhages in his paternal line; his paternal grandmother had died of a ruptured abdominal aortic aneurysm, and his paternal great-grandmother had died of a brain hemorrhage. The patient was not known to have any personal or family history of connective tissue disorders and did not have any overt features typical of an underlying connective tissue disorder, such as a marfanoid habitus.

Following the patient’s discharge, he underwent a series of outpatient investigations. Three-lead event recording over five days did not reveal any paroxysmal arrhythmia. A transthoracic echocardiogram was unremarkable. The bubble echocardiogram did not reveal any intra-atrial communication.

The patient underwent extensive community-based rehabilitation. When reviewed in the clinic approximately one year after discharge, the patient had some ongoing mild right-sided limb weakness but had returned to working part-time from home. The patient had also stopped smoking, drinking alcohol, and using cocaine. He had not suffered any further stroke or known dissection.

In the nine months following this patient’s discharge from the hospital, he was treated twice for a lower limb deep vein thrombosis (DVT), with a three-month course of oral anticoagulation with apixaban, followed by a lifelong course after his second event. He had not suffered any known thromboembolic event prior to his stroke. The patient was referred to our hematology colleagues, who are organizing genetic testing and will also review the indication to continue lifelong anticoagulation. In our hematology colleague’s opinion, the DVTs were likely provoked by relative immobility following this man’s stroke. Although the patient had a strong family history of hemorrhages, there was no known history of thromboembolism.

## Discussion

We presented a man who presented with a left MCA infarct, simultaneous non-traumatic spontaneous intracranial and extracranial arterial dissection, and two DVTs occurring in the following nine months. Although CAD has been reported to occur simultaneously in more than one artery in around 15-20% of cases, this is, to our knowledge, the only reported case of multiple dissections of the same cervicocephalic artery.

The factors that may have led to our patient’s unusual combination of two dissections in the same artery are not easily elucidated. CAD can be triggered by a variety of mechanical triggers, such as blunt neck trauma or cervical hyperextension, but our patient reported no such experience. It is generally considered that "spontaneous" CADs, such as this one appears to be, arise when there is an underlying arterial wall weakness, which allows a dissection to be triggered by an apparently trivial trauma that may go unnoticed at the time. The patient’s family history of different types of hemorrhage causing death might be suggestive of a genetic predisposition to arteriopathy, but the significance of this in relation to the CADs is unclear. Evidence for the heritability of CAD is not strong: in a large 2014 study, for example, only 1% of patients with spontaneous dissections had a family history of CAD [10].

Conditions that are known to increase the risk of CAD do include some genetic connective tissue disorders, particularly vascular Ehlers-Danlos syndrome (EDS): in one study, over 40% of patients with confirmed vascular EDS sustained a CAD by the time they reached age 40 [11], although the rarity of this condition means that it can only be associated with less than 2% of reported CAD cases [12]. Our patient showed no phenotypic features characteristic of EDS or of other hereditary connective tissue disorders, such as Marfan’s syndrome, osteogenesis imperfecta, or cystic medial necrosis, which have also been linked to CAD in some cases. There is growing evidence, however, to support the hypothesis that a range of genetically determined subclinical connective tissue disorders may be involved in predisposition to CAD [9]. Abnormalities of collagen and fiber networks were frequent findings in patients with spontaneous CAD: 50.5% of the cumulative 295 patients from 10 studies showed such connective tissue aberrations. These ultrastructural changes were observed in the absence of known clinical manifestations of connective tissue disorders.

Whole-exome and whole-genome studies, as well as proteomics methods, have identified several genetic variations and abnormal protein expression patterns associated with connective tissue assembly in CAD patients. It seems likely that genetic predisposition to CAD is rarely a monogenic phenotype but rather the result of a complex and variable combination of factors.

Other clinically significant conditions that have been shown to have significant associations with CAD include fibromuscular dysplasia (FMD); one study found that nearly 40% of CAD patients had FMD [13]. The etiology of this arterial wall abnormality is poorly understood, but there is strong evidence for a degree of heritability, and, interestingly, abnormalities in the same gene have been linked to both CAD and FMD in at least one case [14]. The association with FMD is even stronger in patients with multiple dissections [15]. However, there was no clear evidence of FMD in our patient’s case.

We cannot, therefore, identify a specific likely underlying cause for the double CAD observed in this case. The patient's heavy smoking is a known risk factor for CAD [16], and his family history of hemorrhage could be indicative of a potentially relevant hereditary arteriopathy, but there is nothing definitive in the available evidence to suggest why even a single CAD would have occurred.

Cocaine use is associated with cervicocephalic dissection [17]. The pathogenesis of this is thought to be multifactorial, with cocaine’s sympathomimetic properties leading to arterial vasoconstriction, hypertension, and increased shear stress on the vasculature, potentiating arterial endothelial damage that could lead to dissection formation. It is possible that the general weakening of the arterial wall as a result of the use of these substances may be a reason for the occurrence of multiple dissections in contrast to cases where the cause is a trauma, causing damage at a single, specific position site, but there is no clear evidence of this.

We note that in the present case, the two separate ICA dissections, although observed simultaneously by angiography, may not have occurred simultaneously, as CAD can in some instances be asymptomatic and the healing time is typically several months [18]. It is possible, therefore, that this might be a case of recurrent CAD, where the initial lesion caused no clear clinical symptoms but later, the second one precipitated the stroke. Epidemiological studies of the recurrence of CAD have shown that this is rare, occurring in only around 4% of CAD cases [19], and it is generally observed to occur in different arteries rather than within the same artery [20]. Again, therefore, our patient would appear to be an unusual case.

## Conclusions

Spontaneous CAD is an uncommon cause of stroke in the entire patient cohort but a relatively common cause of stroke in younger stroke patients. Multiple CADs are rare, and to our knowledge, this is the first report of multiple dissections of the same artery.

Various risk factors for CAD have been identified, including FMD and EDS, neither of which was evident in this patient. There is growing evidence that genetically determined subclinical connective tissue disorders are associated with CAD, but we cannot tell if any of these here. The patient’s lifestyle choices, including smoking and cocaine use, would certainly have increased the risk of CAD in general, but there is nothing in his history that might have been expected to lead to the unusual combination of dissections that occurred.
